# Low socioeconomic status predicts vitamin D status in a cross-section of Irish children

**DOI:** 10.1017/jns.2022.57

**Published:** 2022-07-25

**Authors:** Helena Scully, Eamon Laird, Martin Healy, Vivion Crowley, James Bernard Walsh, Kevin McCarroll

**Affiliations:** 1 Mercers Institute for Research on Ageing, St James's Hospital, Dublin 8, Ireland; 2 School of Medicine, Trinity College Dublin, Dublin 2, Ireland; 3 Department of Biochemistry, St James's Hospital, Dublin 8, Ireland

**Keywords:** 25(OH)D, Childhood, Ireland, Socioeconomic status, Vitamin D, Vitamin D deficiency

## Abstract

Vitamin D is essential for bone and muscle health with adequate status in childhood crucial for normal skeletal development. We aimed to investigate vitamin D status in a convenience sample (*n* = 1226) of Irish children (aged 1–17 years) who had serum 25-hydroxyvitamin D (25(OH)D) tested by request of their GP at a Dublin Hospital between 2014 and 2020. We examined predictors including age, sex, season and socioeconomic status (SES). Vitamin D deficiency (<30 nmol/l) was prevalent affecting 23 % and was more common in disadvantaged areas (34 %) and in those aged >12 *v*. ≤12 years (24 % *v*. 16 %, *P* = 0⋅033). The greatest predictor was SES (disadvantaged *v*. affluent, OR 2⋅18, CI 1⋅34, 3⋅53, *P* = 0⋅002), followed by female sex (OR 1⋅57, CI 1⋅15, 2⋅14, *P* = 0⋅005) and winter season (October to February, OR 1⋅40, CI 1⋅07, 1⋅84, *P* = 0⋅015). A quarter of our sample of children were deficient, rising to one-third in those in disadvantaged areas. Females and those aged over 12 years had a higher prevalence of deficiency. Public health strategies to improve vitamin D status in Irish children, including systematic food fortification may need to be considered to address this issue.

## Introduction

Vitamin D (cholecalciferol) is a secosteroid produced via the action of Ultraviolet-B (UVB) light on skin. It is essential for the adequate absorption of calcium from the gastrointestinal tract and normal skeletal development^(1)^. Vitamin D deficiency (25 hydroxyvitamin D (25(OH)D) <30 nmol/l) in children can lead to impaired bone mineralisation causing rickets or osteomalacia^(1)^. Concernedly, a case study of Irish infants in 2006 suggests a rise in rickets incidence^(2)^, as found in the UK, where cases have reached a 50-year peak^(3)^. While the overall prevalence of rickets is low, those at increased risk include young children under 5 particularly those of Black and South-east Asian ethnicity^(4)^.

Importantly, peak bone mass in adolescence and early adulthood is influenced by vitamin D status and may account for 60 % of osteoporosis risk in later life^(5)^. Vitamin D may also have a role developmentally in early life consistent with the Barker fetal origins hypothesis^(6)^. For example, it may be important in fetal epigenetic programming of respiratory conditions in childhood^(7)^ and has been associated with language and motor skill development, and risk of autism and ADHD^(8,9)^. Deficiency in childhood has been associated in some studies with extra-skeletal diseases^(9)^ such as hypertension^(10)^, diabetes^(11,12)^, depression^(13)^, dental caries^(14)^, atopy and asthma^(15,16)^. Additionally, vitamin D might support childhood immune function with evidence suggesting a protective effect against respiratory tract infections. However, randomised control trials are required to further explore these associations^(17)^.

Due to Irelands’ north latitude (53°N), little or no vitamin D can be synthesised during winter months and adult deficiency is prevalent^(18,19)^. However, studies in Irish children and adolescents are limited, with deficiency (<30 nmol/l) ranging between 5 and 22 % and levels <50 nmol/l reported to be between 27 and 89 %^(20–23)^. Factors related to familial socioeconomic status (SES) have been suggested to affect vitamin D including lower diet quality, reduced intake of oily fish and supplement intakes and less access to outdoor amenities^(24–26)^. While some studies in Ireland have looked at the predictors of vitamin D, none have investigated any relationship with SES in children. In the UK, social deprivation and lower household income has been found to be independently associated with childhood vitamin D deficiency^(27,28)^.

Given the limited studies in Ireland, we aimed to assess the association of SES and vitamin D status in children (1–17 years) in the Dublin area and surrounds who had 25(OH)D levels tested at our hospital laboratory by request of their General Practitioner (GP). In addition, we aimed to identify the prevalence of deficiency and its variation by age, sex and season. We also examined the proportion of children who were retested and factors associated with this.

## Methods

### Data collection

Data were collected at St James's Hospital (SJH), Dublin, Republic of Ireland (53° Northern Latitude). It serves a population of approximately 350 000 and receives referrals primarily from Dublin city and its surrounds. Using the laboratory information system (iSOFT Telepath®) at SJH Biochemistry Department, a search was completed for vitamin D requests from primary care GPs between 2014 and 2020. A convenient sample was identified using the following exclusion criteria: age ≥18 years on initial test, incomplete or missing demographic data, non-community address (e.g. Hospital) or location outside the Republic of Ireland. We also identified any repeat vitamin D tests (i.e. retests) for each participant during the study period.

### Socioeconomic status

Participant socioeconomic status (SES) was assessed by mapping postal addresses using the 2016 Pobal HP (Haase-Pratschke) Deprivation Index^(29)^. This is a composite score of demographic profile, social class composition and labour market situation of small areas based on the 2016 Census of the Population. Small areas (100 households mean) are given a Relative Index Score that is then categorised into the following groups: extremely disadvantaged (≤−30), very disadvantaged (−20 to −29⋅99), disadvantaged (−10 to −19⋅99), marginally below average (0 to −9⋅99), marginally above average (0–9⋅99), affluent (10–19⋅99), very affluent (20–29⋅99) and extremely affluent (≥30). In this analysis, categories were combined into four groups: (1) disadvantaged (extremely disadvantaged, very disadvantaged, disadvantaged), (2) below average, (3) above average and (4) affluent (extremely affluent, very affluent, affluent), as previously described^(30)^.

### Ethics

Ethical approval for this study was granted by the St James's Hospital/Tallaght University Hospital (SJH/TUH) joint ethics committee (Ref: 5475). This study was conducted according to the Declaration of Helsinki 1964 guidelines.

### Serum 25(OH)D and biochemical markers

Vitamin D (total 25(OH)D2 and 25(OH)D3) was measured using liquid chromatography tandem mass spectrometry (LC-MS/MS) (API 400; AB SCIEX) at the Biochemistry Department of SJH. A validated method (Chromsystems Instruments and Chemicals GmbH; MassChrom 25-OH-Vitamin D3/D2) accredited to ISO 15189:2012 standards was employed for analysis. Assay quality was ensured by participation in the vitamin D External Quality Assessment Scheme (DEQAS) and assay of internal and third-party quality controls and has been accredited by the Irish National Accreditation Board (INAB). Our vitamin D results fall within 3⋅5 % of the target control value, and this was similar for all years during the study. Accuracy was determined using the National Institute of Standards and Technology (NIST) 972 25(OH)D standard reference material (SRM 972). The respective inter- and intra-assay coefficients of variation are 5⋅7 and 4⋅5 %. In this study, we defined deficiency: <30 nmol/l, insufficiency: 30⋅0–49⋅9 nmol/l and sufficiency: ≥50 nmol/l^(31)^. Participants with 25(OH)D level >125 nmol/l were also identified as there may be associated health risks above this concentration^(31)^. Winter was defined as (October–February); and summer (March–September) as used elsewhere^(32)^.

### Statistics

Statistical analysis was carried out using SPSS (Version 26, IBM Corp., Armonk, NY, USA). The population was dichotomised by age (≤12 or >12 years) as in previous studies^(33–35)^. Data were checked for normality by the Kolmogorov–Smirnov test. Geometric mean with standard deviation was reported in tables. Categorical variables were tested using *χ*^2^, with independent sample *t*-tests, one-way ANOVA and Kruskal–Wallis test for continuous variables. Statistical significance was accepted when *P* < 0⋅05. Independent factors associated with vitamin D deficiency were explored in multi-nominal logistic regression models using the following variables: age category, sex, socioeconomic status and season of sampling. In a similar model, we explored for predictors of vitamin D retesting.

## Results

Demographics of the cohort are shown in Table 1. Vitamin D results (not including retests) were initially identified for 1294 children aged between 1 and 17 years. After exclusion of participants with no available address (*n* = 25), and in whom SES could not be mapped (*n* = 43) the final number was 1226. The majority (69⋅2 %) were female and 89⋅3 % were aged >12 years. A similar proportion was tested in summer (56⋅5 %) and winter (43⋅5 %). The most prevalent SES classification was above average (43⋅6 %), followed by affluent (37⋅5 %), below average (11⋅1 %) and disadvantaged (7⋅8 %). The number tested was lower in 2014/2015 with annual increases thereafter in the period up to 2019. Nearly one-fifth (17⋅6 %, *n* = 228) of participants had vitamin D retested in the study period.

**Table 1.** Population demographics

Category		*n*	%
Age	≤12 years	131	10⋅7
	>12 years	1095	89⋅3
SES	Affluent	460	37⋅5
	Above Average	534	43⋅6
	Below Average	136	11⋅1
	Disadvantaged	96	7⋅8
Season	Winter	533	43⋅5
	Summer	693	56⋅5
Sex	Female	848	69⋅2
	Male	378	30⋅8
Year	2014	74	6⋅0
	2015	103	8⋅4
	2016	191	15⋅6
	2017	188	15⋅3
	2018	214	17⋅5
	2019	241	19⋅7
	2020	215	17⋅5
Total		1226	

Overall, 23 % were vitamin D deficient, ranging from 20⋅1 % in summer to 25⋅9 % in winter (Table 2). More than half (50⋅6 %) had levels below 50 nmol/l, which was more common in winter compared to summer (55⋅3 % *v*. 46⋅9 %, *P* = 0⋅003). Mean 25(OH)D was higher in those aged under *v*. older 12 years (43⋅3 nmol/l *v*. 49⋅5 nmol/l, *P* = 0⋅020). It was also lower in females *v*. males (42⋅3 nmol/l *v*. 47⋅6 nmol/l, *P* = 0⋅008) but the difference was only significant in winter (39⋅4 nmol/l *v*. 45⋅7 nmol/l, *P* = 0⋅021). Serum 25(OH)D also varied by year ranging from an average of 39⋅1 nmol/l in 2018 to 50⋅7 nmol/l in 2014 (*P* < 0⋅001).

**Table 2.** Serum 25(OH)D status and concentration by age and sex (dichotomised by season)

Category	*n*	Total	*P*-value	*n*	Winter	*P*-value	*n*	Summer	*P*-value
25(OH)D	1226	43⋅9 (25⋅3)		533	41⋅2 (24⋅4)		693	46⋅1 (25⋅7)	<0⋅001*
<30 nmol/l (%)	277	22⋅6		138	25⋅9		139	20⋅1	0⋅015*
<50 nmol/l (%)	620	50⋅6		295	55⋅3		325	46⋅9	0⋅003*
Sex									
Female	848	42⋅3 (25⋅3)	0⋅008	376	39⋅4 (24⋅8)	0⋅021	472	44⋅8 (25⋅5)	0⋅148
Male	378	47⋅6 (25⋅0)		157	45⋅7 (23⋅1)		221	49⋅0 (26⋅2)	
Age									
≤12 years	131	49⋅5 (25⋅4)	0⋅020	56	46⋅8 (21⋅3)	0⋅053	75	51⋅6 (27⋅9)	0⋅147
>12 years	1095	43⋅3 (25⋅2)		477	40⋅5 (24⋅7)		618	45⋅5 (25⋅4)	

25(OH)D reported as Geometric mean ± (standard deviation) in nmol/l.

* *P*-value for winter season (October–February) *v*. summer season (March–September). *P*-values are reported for within category differences, using Mann–Whitney *U* or *χ*^2^ test.

**Fig. 1.**
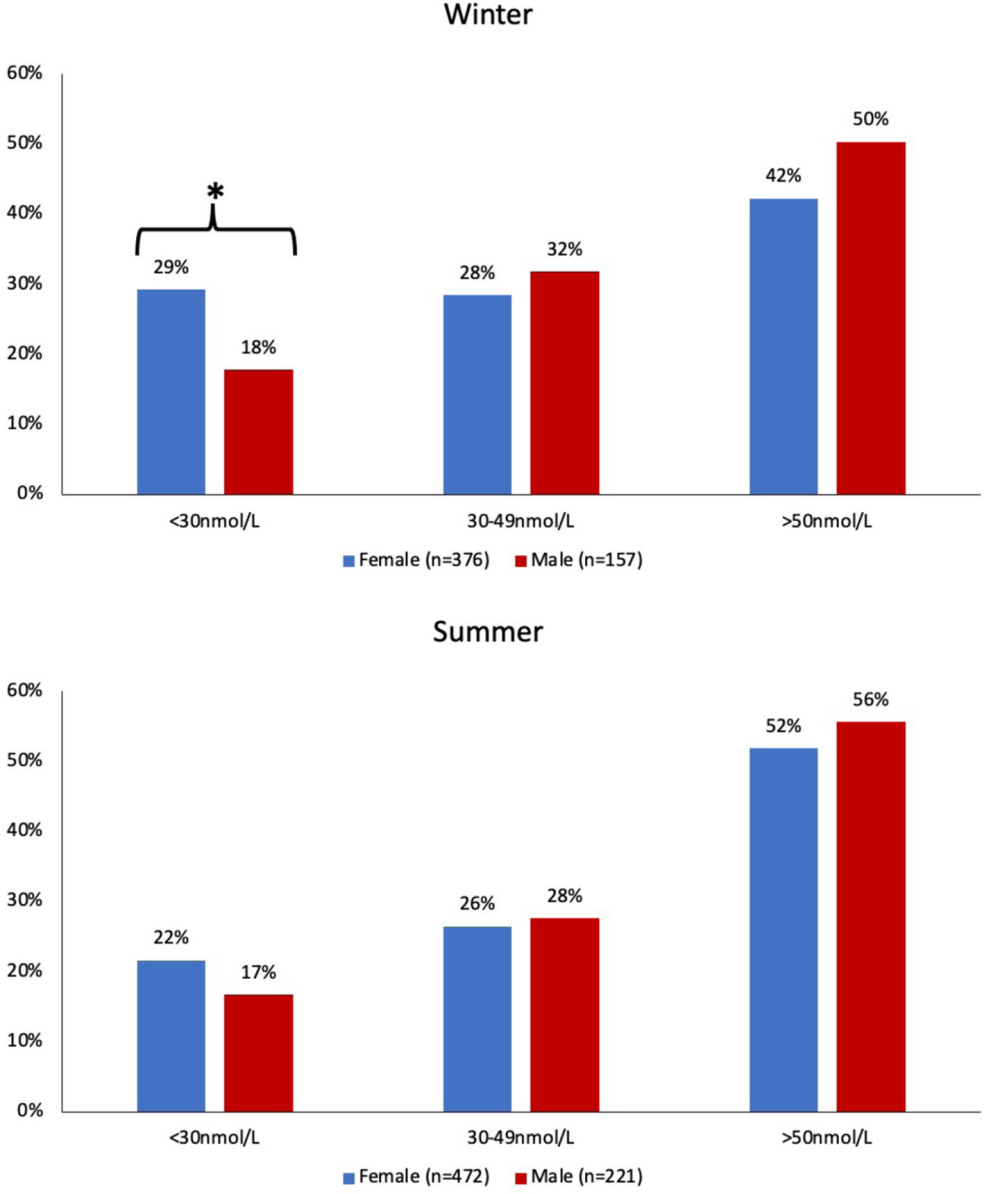
Vitamin D status by sex. *Indicates significance (*P* < 0⋅05) analysed by *χ*^2^. Winter (October–February); Summer (March–September).

Females had a higher prevalence of deficiency *v*. males (25 % *v*. 17 %, *P* = 0⋅003) but this remained significant only in winter (29 % *v*. 18 %, *P* = 0⋅006) (Fig. 1). There was also a lower proportion of females *v*. males who were vitamin D sufficient (≥50 nmol/l) in both seasons but this was not statistically significant. The overall prevalence of 25(OHD) >125 nmol/l was 0⋅6 % with no difference by season or sex.

**Fig. 2.**
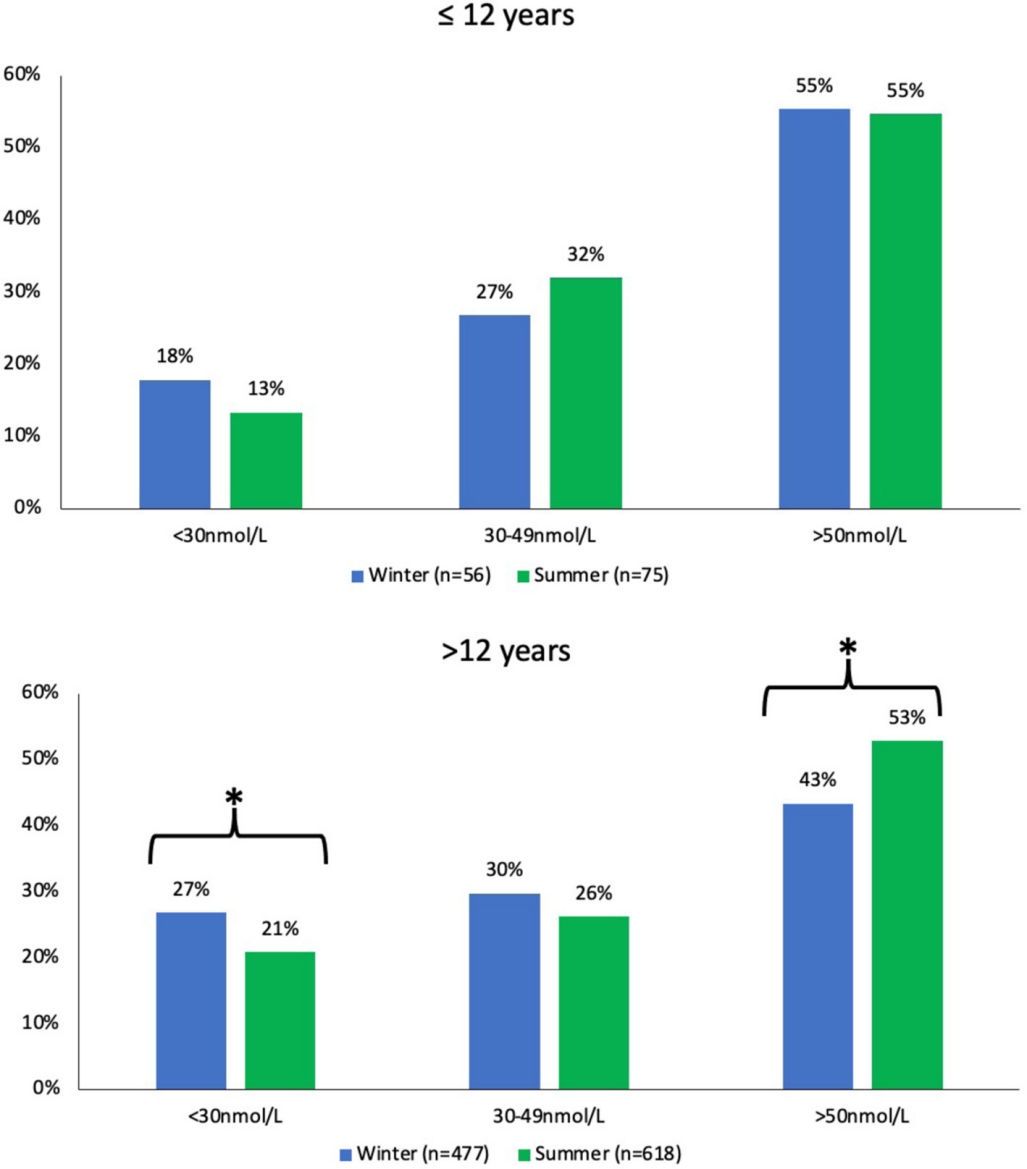
Vitamin D status by age. *Indicates significance (*P* < 0⋅05) analysed by *χ*^2^. Winter (October–February); Summer (March–September).

Vitamin D deficiency was more prevalent in those aged over *v*. under 12 years (23 % *v*. 15 %, *P* = 0⋅034). In the over 12s, deficiency was also greater in winter *v*. summer (27 % *v*. 21 %, *P* = 0⋅021) (Fig. 2). Those aged over 12 years had more sufficiency in summer *v*. winter (53 % *v*. 43 %, *P* = 0⋅002). We also found more deficiency in those ≤12 years in summer *v*. winter but this was not statistically significant. The prevalence of serum 25(OH)D >125 nmol/l was 0⋅7 and 0⋅5 % in the under and over 12s. Overall, levels of deficiency were significantly higher (26 % *v*. 20 %, *P* = 0⋅015), and sufficiency significantly lower (45 % *v*. 53 %, *P* = 0⋅003), in winter *v.* summer.

**Fig. 3.**
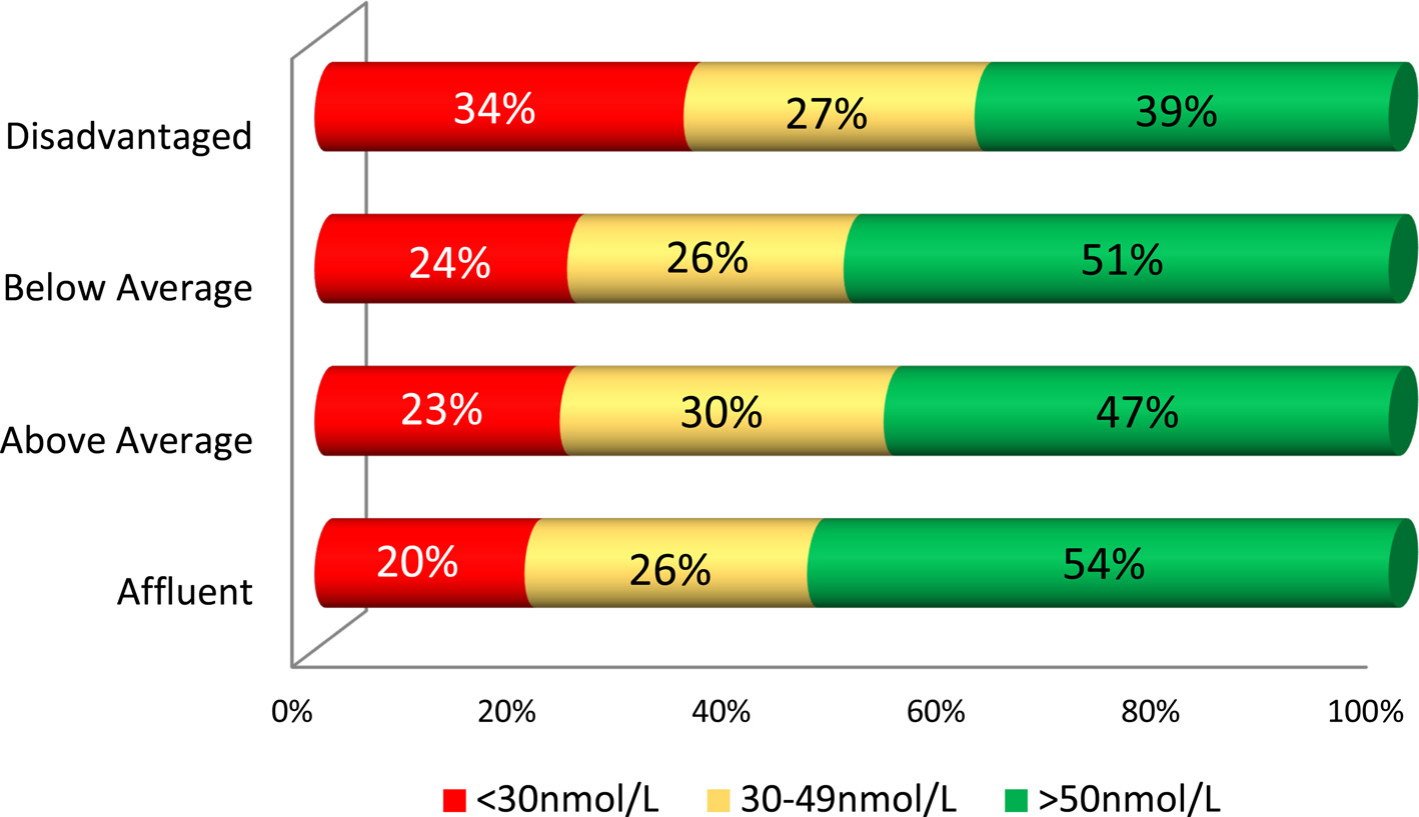
Vitamin D status by socioeconomic status.

Vitamin D status and concentration by socioeconomic category are presented in Table 3. The difference in vitamin D status was greatest between affluent and disadvantaged areas. Children in below or above average areas had intermediate vitamin D levels with little difference between both of these SES categories, though being higher than disadvantaged. There were also significant differences in deficiency by area (*P* = 0⋅0018) ranging from 34 % in disadvantaged to 20 % in affluent areas. Similarly, the lowest prevalence of levels below 50 nmol/l was identified in affluent compared to disadvantaged areas (46 % *v*. 61 %, *P* = 0⋅019) (Fig. 3). When comparing below and above average SES, there was no difference in the prevalence of deficiency (*P* = 0⋅866) or levels between 30 and 49 nmol/l (*P* = 0⋅312). There were no significant differences in serum 25(OH)D in females (*P* = 0⋅051) or males (*P* = 0⋅127) across SES categories but sample sizes were smaller. In both those over and under 12 years, there was a significant difference in vitamin D status (*P* < 0⋅05) by SES with higher levels in affluent areas. Summertime vitamin D status was also significantly different across SES categories (*P* = 0⋅010) and highest in affluent areas.

**Table 3.** Serum concentration 25(OH)D by socioeconomic status category

nmol/l	*n*	Socioeconomic status category				*P*-value
		Disadvantaged	Below average	Above average	Affluent	
Overall	1226	38⋅1 (23⋅7)	43⋅0 (26⋅6)	43⋅1 (25⋅5)	46⋅5 (24⋅7)	0⋅005*
<30 (%)	227	34	24	23	20	0⋅018*
<50 (%)	343	61	49	53	46	0⋅019*
Sex						
Male	378	37⋅7 (24⋅2)	47⋅5 (25⋅1)	47⋅3 (26⋅0)	50⋅4 (23⋅8)	0⋅127
Female	848	38⋅2 (23⋅7)	41⋅3 (27⋅2)	41⋅4 (25⋅1)	44⋅8 (25⋅1)	0⋅051
Age (years)						
≤12	131	36⋅9 (14⋅9)	57⋅6 (19⋅6)	44⋅3 (27⋅4)	56⋅2 (24⋅0)	0⋅031*
>12	1095	38⋅1 (24⋅1)	41⋅2 (27⋅2)	42⋅9 (25⋅3)	45⋅5 (24⋅7)	0⋅020*
Season						
Winter	533	37⋅3 (21⋅5)	39⋅5 (27⋅9)	40⋅6 (24⋅6)	43⋅0 (23⋅7)	0⋅271
Summer	693	38⋅6 (25⋅3)	45⋅7 (25⋅6)	44⋅9 (26⋅0)	49⋅8 (25⋅2)	0⋅010*

25(OH)D reported as Geometric mean ±(standard deviation) in nmol/l. Winter (October–February); Summer (March–September). *P*-values are reported for within category differences, using *χ*^2^ or Kruskal–Wallis test.

* Indicates significance at <0⋅05 level.

Predictors of vitamin D deficiency are shown in Table 4. The greatest predictor was living in a disadvantaged location (OR 2⋅18, CI 1⋅34, 3⋅53, *P* = 0⋅002), followed by female sex (OR 1⋅57, CI 1⋅15, 2⋅14, *P* = 0⋅005), and testing in the winter (OR 1⋅40, CI 1⋅07, 1⋅84, *P* = 0⋅015). Age was not an independent predictor, though the number under 12 years was small (*n* = 139) and the analysis was likely to be underpowered to detect a significant difference.

**Table 4.** Predictors of vitamin D deficiency (in multinomial regression)

Deficient (<30 nmol/l) *v*. non-deficient (≥30 nmol/l)	*n*	B	OR	Confidence Interval		*P*-value
				Lower	Upper	
Intercept	1226	−1⋅863				
Age ≤12 years	131	−0⋅47	0⋅625	0⋅379	1⋅032	0⋅066
Age >12 years^†^	1095					
Disadvantaged	96	0⋅778	2⋅176	1⋅34	3⋅534	0⋅002*
Below Average	136	0⋅256	1⋅292	0⋅814	2⋅052	0⋅277
Above Average	534	0⋅222	1⋅249	0⋅917	1⋅702	0⋅159
Affluent^†^	460					
Female	848	0⋅451	1⋅569	1⋅149	2⋅144	0⋅005*
Male^†^	378					
Winter	533	0⋅338	1⋅402	1⋅069	1⋅840	0⋅015*
Summer^†^	693					

B, unstandardised beta, OR, odds ratio.

* Indicates significance at <0⋅05 level.

† Indicates reference variable. Winter (October–February); Summer (March–September).

Predictors of vitamin D retesting are shown in Supplementary Table S1. Baseline deficiency was the greatest predictor (OR 1⋅77, CI 1⋅22, 2⋅56, *P* = 0⋅003), followed by insufficiency (OR 1⋅76, CI 1⋅24, 2⋅49, *P* = 0⋅002) and female sex (OR 1⋅45, CI 1⋅03, 2⋅03, *P* = 0⋅032). There was a trend for reduced testing in those living in disadvantaged (OR 0⋅55, CI 0⋅29, 1⋅05, *P* = 0⋅068) and above average locations (OR 0⋅72, CI 0⋅52, 1⋅00, *P* = 0⋅052).

## Discussion

This is the largest investigation of vitamin D status in a convenience sample of Irish children and adolescents and the only one to explore the association with SES. We identified that 23 % overall were vitamin D deficient (<30 nmol/l), rising to 34 % in children living in disadvantaged areas. In total, half of the cohort had concentrations less than 50 nmol/l, indicating that inadequate vitamin D status is highly prevalent. The greatest predictor for deficiency was living in disadvantaged locations, followed by female sex, and testing in the winter (October–February). Those over 12 also had lower vitamin D status than those under 12 years. We also showed that about one in five children were retested which is similar to that found in Irish adults^(36)^, with predictors of retesting including initial deficiency/insufficiency and female sex.

### Vitamin D status by socioeconomic status

Children living in disadvantaged areas were more than twice as likely to be vitamin D deficient compared to affluent children. This is the first study in Ireland examining the effect of SES on vitamin D status in children. The only other Irish study investigating vitamin D status and SES was in older adults as part of the TILDA study, which found that those with below average asset wealth had 1⋅5 times increased prevalence of vitamin D deficiency^(19)^. In the UK, low socioeconomic status has also been associated with greater deficiency^(27,28)^ and similar results have been identified in the Netherlands^(37)^, Greece^(38)^ and Canada^(39)^.

Lower SES may impact on vitamin D status as it has been associated with factors (e.g. reduced physical activity outdoors) that may lower UVB exposure^(40)^, reduced dietary and supplemental vitamin D intake and greater obesity prevalence^(24–26)^. Supplements are a key contributor of vitamin D intake in Irish children and adolescents^(41,42)^. However, low supplement use has previously been found in children in low income or food insecure households^(25)^ and has also been correlated with parental education^(43)^. Total vitamin D intake has been identified as being lower in children in lower income families in the UK and Spain^(44,45)^. This may be due to an increased prevalence of unhealthy eating in low SES children with lower consumption of vitamin D-rich foods including oily fish, meat and fortified food^(24,46,47)^. Furthermore, lower serum 25(OH)D has been found in Canadian children (*n* = 1753) from lower income families^(48)^. Studies of Irish adults have also found an association with disadvantaged backgrounds and a lower likelihood of meeting dietary vitamin D intake recommendations^(49)^ with lower consumption of foods that are typical sources of vitamin D including fish, meat and breakfast cereals. In the US, vitamin D intake in children and adults was also correlated with income, with greater levels shown in the highest income group^(50,51)^.

Another factor that may help explain vitamin D status by SES group is differences in rates of obesity. For example, obesity or overweight status in Irish children was more prevalent in those attending disadvantaged schools^(52)^. Obesity is associated with lower vitamin D in children, possibly due to its sequestration in adipose tissue^(53)^. We also identified seasonal differences in vitamin D status across SES categories, with those in affluent areas having higher serum levels in summer. This could be related to increased sun holiday travel, which is a reported determinant of vitamin D status in Irish adults^(54)^. Additionally, higher socioeconomic status is associated with reduced screen-time, increased access to outdoor activities and engagement in organised sports in adolescents, resulting in increased sun exposure^(26,55,56)^. In deprived areas, there may be reduced access to parks/playgrounds/gyms, which could also lower UVB exposure^(56,57)^.

### Vitamin D status by age

We identified that children aged >12 years had a greater prevalence of deficiency consistent with other studies in Europe, the US and Asia^(27,58–60)^. The only other Irish study comparing vitamin D status by age category was small (*n* = 252) and found a lower mean 25(OH)D in 12–17 years old *v*. those aged 1–4 years^(20)^. A larger Irish study of toddlers (aged 2 years) found a prevalence of deficiency (4⋅6 %) about five times lower than in the over 12s in our study^(22)^. Similarly, in the most recent UK National Diet and Nutrition Survey, deficiency was greater in those aged 11–18 (19 %) *v*. 4–10 years (2 %)^(61)^. In Europe, higher rates of deficiency (<30 nmol/l) were found in teenagers *v.* younger children^(58)^. Likewise, in a large US study (*n* = 16 180), over 12s had the greatest prevalence of serum 25(OH)D (<50 nmol/l)^(62)^. Lower vitamin D status in older children may reflect reduced intake of vitamin D fortified foods, more sedentary behaviour or screen-time and higher rates of obesity^(33,35,60)^. Indeed, a dietary survey (*n* = 594) of Irish children and teens found a lower rate of supplement use and fortified foods in adolescents (13–17 years) *v*. younger children (9–12 years)^(41)^. Increased vitamin D requirements in adolescence^(63)^ due to an intensive period of new bone growth has also been proposed as a factor^(31)^. Finally, greater diagnosis of deficiency in older children might be in part due to more frequent presentation to GP's for chronic pain/medical symptoms or higher thresholds for vitamin D tests in younger children due to the challenges of phlebotomy^(28)^.

### Vitamin D status by season and sex

As expected, there was a seasonal variation in 25(OH)D, consistent with most studies^(20,35,39,40)^, with more deficiency in winter *v.* summer. Furthermore, 55⋅5 % of children had a 25(OH)D below 50 nmol/l in winter. This figure is identical (55⋅3 %) to the recent finding in children (*n* = 47, age 7–11) living in Northern Ireland between November and March^(21)^. While the prevalence of deficiency/insufficiency varied by season in the under 12s, the difference was not significant, but is likely explained by the small sample size and lack of statistical power. A notable finding was the lower vitamin D status in females who were more likely to be deficient, a finding similar to other studies in Europe and elsewhere. For example, deficiency has been reported to be more common in Northern Irish female adolescents^(64)^, female children in Britain^(1,27)^, Greece^(65)^ and the US^(60)^. Despite this, another study in Germany identified more deficiency in males^(66)^.

We did find though that the difference in 25(OH)D status by sex was only significant in winter, albeit with a trend for better vitamin D status in males in summer. Dietary intake may be a contributory factor as female children have been found to have a lower vitamin D intake and consume less vitamin D fortified foods^(67–69)^. Body composition during puberty may play a role, with females acquiring greater fat mass during maturation^(70)^. Additionally, it is possible that adolescent girls may engage more in veganism, as found in female adults^(71)^, which could lower vitamin D intake due to avoidance of meat and milks, that are significant sources of dietary vitamin D^(35)^. Females within ethnic minorities may also have reduced exposure due to religious dress^(26)^. On the other hand, male adolescents are reported to engage in more outdoor activity which affords more opportunity for sun exposure^(27,64)^. They are also less likely to use sunscreen or take measures to avoid sunburn^(72)^ and may have more cutaneous exposure to summer UVB due to less clothing cover^(27,73)^. Finally, it has been suggested that higher overall GP consultation rates for girls than boys might account in part for a greater diagnosis of deficiency^(28,74)^.

### Guidelines on vitamin D intakes and implications

The Food Safety Authority of Ireland (FSAI) advise a vitamin D intake of 5 μg/d for children aged 1–5 years^(2)^ though have only a blanket recommendation of 10 μg/d for those aged 5–65 years^(75)^. Guidelines are similar in the UK, with an intake (RNI) of 10 μg/d for those aged >4 years and a ‘Safe intake’ of 10 μg/d between 1 and 4 years^(1)^. However, the European Food Safety Authority (EFSA) and the US Institute of Medicine (IOM) recommend a higher dietary allowance of 15 μg/d between 1 and 18 years^(75,76)^. Despite more conservative guidelines in Ireland, 70–84 % of 1–4-year olds^(34)^ and 94 % of 5–18-year olds have inadequate vitamin D intakes^(33,35)^.

Indeed, it has been suggested that vitamin D intakes for children may need to be substantially higher. For example, an estimated total vitamin D intake (dietary and supplemental) of 33⋅8 μg/d may be required for 97⋅5 % of children living at 40–63°N to be vitamin D sufficient (>50 nmol/l) in the winter^(77)^. However, guidelines alone may not improve vitamin D status. In Canada, after dietary guidelines for vitamin D intake increased from 5 to 15 μg daily for those aged 1–70 years, an actual increase in vitamin D insufficiency (<50 nmol/l) was identified in those aged 6–18 years^(78)^. Targeted systematic vitamin D fortification of food is another option, as occurred in Finland in 2003. However, while it led to 91 % of over 30s in the population achieving 25(OH)D >50 nmol/l^(79)^, it produced little improvement in vitamin D intake or serum levels in adolescent females^(67)^ who had lower fortified food consumption^(80)^. The significant prevalence of vitamin D deficiency in our cohort of children suggests that vitamin D intakes are inadequate for a sizeable proportion of those aged 1–17 years, particularly in disadvantaged areas. Guidelines on vitamin D intakes that are specifically tailored to include all Irish children should be developed. However, as studies show these measures alone tend to be inadequate, other strategies including targeted vitamin D fortification of foods that are consumed by children needs to be considered.

### Strengths

This is the largest study of vitamin D status in Irish children (1–17 years), and the first to investigate the association with socioeconomic status. We used a specific measure of SES, that is localised to a small area of 100 households, whereas most other studies used proxy measures such as parental education or household income. We used the gold standard of vitamin D measurement, LC-MS and adhere to strict quality monitoring (participation in DEQAS). We also utilised a dataset collected over a 7-year period that included and also explored for predictors of retesting.

### Limitations

The present study is based on a convenient sample of GP vitamin D requests which limits the generalisability of the findings to a wider population. In particular, there may be selection bias of study participants who may have conditions predisposing to vitamin D deficiency that underlies the reason for their testing. We were not able to account for factors that influence serum 25(OH)D including biophysical (ethnicity, body mass index, medical conditions) or lifestyle (dietary/supplement intake, sun exposure or sunscreen use) due to the nature of the collected data. Finally, as the study is cross-sectional, we cannot infer any causality with regard to the factors we examined.

## Conclusion

In conclusion, we identified that in a large convenience sample of children attending their GP in Ireland, those in the most disadvantaged area had the highest level of deficiency, affecting 34 %. Furthermore, about one quarter of all children were found to be deficient. Childhood and adolescence are crucial periods for bone and muscle development, and deficiency may have long-term effects on both skeletal and other health outcomes. Targeted and tailored guidelines on vitamin D intake for all Irish children as well as public health promotion of its importance should be a priority. Development of a systematic policy of a vitamin D fortification of foods regularly consumed by children could be a realistic approach to help mitigate this issue.

## Supplementary material

For supplementary material accompanying this paper visit https://doi.org/10.1017/jns.2022.57. click here to view supplementary material
